# Expression, Purification, and Preliminary Protection Study of Dehydrin PicW1 From the Biomass of *Picea wilsonii*

**DOI:** 10.3389/fbioe.2022.870672

**Published:** 2022-04-05

**Authors:** Junhua Liu, Mei Dai, Jiangtao Li, Yitong Zhang, Yangjie Ren, Jichen Xu, Wei Gao, Sujuan Guo

**Affiliations:** ^1^ Biological Physics Laboratory, College of Science, Beijing Forestry University, Beijing, China; ^2^ National Engineering Laboratory of Tree Breeding, Beijing Forestry University, Beijing, China; ^3^ Key Laboratory of Forest Cultivation and Conservation, Ministry of Education, Beijing Forestry University, Beijing, China

**Keywords:** dehydrin, expression, purification, lactate dehydrogenase, protective effects

## Abstract

Dehydrins (DHNs) belong to group II of late embryogenesis-abundant (LEA) proteins, which are up-regulated in most plants during cold, drought, heat, or salinity stress. Despite the importance of dehydrins for the plants to resist abiotic stresses, it is necessary to obtain plant-derived dehydrins from different biomass. Generally, dehydrin PicW1 from *Picea wilsonii* is involved in Kn-type dehydrin with five K-segments, which has a variety of biological activities. In this work, *Picea wilsonii* dehydrin PicW1 was expressed in *Escherichia coli* and purified by chitin-affinity chromatography and size-exclusion chromatography, which showed as a single band by SDS-PAGE. A cold-sensitive enzyme of lactate dehydrogenase (LDH) is used to explore the protective activities of other proteins. Temperature stress assays showed that PicW1 had an effective protective effect on LDH activity, which was better than that of bovine serum albumin (BSA). This study provides insights into the purification and protective activity of K5 DHNs for the advancement of dehydrin structure and function from biomass.

## Introduction

Biomass refers to the organic matter produced by all organisms and growth. Generally, biomass is the abundant renewable resource that can not only be transformed into many high value-added products of chemicals, biofuels, and advanced materials (Dong et al., 2020; Liu et al., 2021; Wang et al., 2021; Xu et al., 2021; Hu et al., 2022) but also contains highly abundant stress-resistant gene resources (Ning et al., 2021). Obtaining proteins from biomass tissues requires extracting genomic DNA and RNA, reverse transcription to obtain cDNA, and then designing specific primers to amplify a specific segment of genomic DNA according to the known similar biomass genes. PCR primers were designed with the recombinant plasmid containing the target gene as the template. The amplified products were recovered and connected to the cloning vector, transformed into *E. coli* and extracted the plasmid, and then the appropriate expression vector was selected. The target protein was successfully obtained through a variety of separation and purification techniques (Zhou et al., 2006). An area of considerable research interest is the plant’s ability to resist a variety of abiotic stresses such as drought, cold, high temperature, and high salinity (Hara et al., 2003; Saavedra et al., 2006; Hundertmark and Hincha, 2008; Hirayama and Shinozaki, 2010). All of these result in cell dehydration (Galau et al., 1986). One family of proteins that is expressed during cell dehydration has been named dehydration proteins (dehydrins, or DHNs) (Close, 1996; Graether and Boddington, 2014). DHNs are highly hydrophilic, and their structural analysis implies that they are intrinsically disordered proteins (IDPs), which provide a flexible property to interact with metal ions and biomolecules (Dunker et al., 2001; Halder et al., 2018; Lv et al., 2018; Gupta et al., 2019). DHNs belong to LEA-DII (late embryogenesis-abundant proteins) family and their types have been classified using the segments K, Y, and S. The K-segment is an approximately 15-amino-acid-long conserved lysine-rich motif, and it tends to adopt an amphipathic α-helix structure according to computational prediction (Lv et al., 2018). The Y-segment is a relatively short segment which is named for the conserved tyrosine residues (Hara et al., 2010). The S-segment is a serine-rich region that can be phosphorylated, and it may play a role in the protein delivery to the nucleus (Goday, 1994). In addition to these segments, Φ-segments (G- and polar amino acid-rich sequences) (Close, 1997), F-segments (Strimbeck, 2017; Wei et al., 2019; Ohkubo et al., 2020), and ChP-segments (Graether and Boddington, 2014) have also been described.

In previous studies, four different proteins from various Spruce species, namely, PicW1, PicW2, PicM, and PicK, were identified and expressed in *E. coli*. It had been proved that PicW genes were an effective antifreeze resource by thermal hysteresis activity and *E. coli* antifreeze tests (Zhao et al., 2017; Zhang et al., 2018). In this study, we report the purification of a protective protein PicW1 from *Picea wilsonii*. PicW1 belongs to the K_5_DHN family, because the protein has five highly conservative QKA segments, and each segment contains 2–5 Lys amino acids. PicW1 was cloned from *Picea wilsonii* and expressed in *E. coli*, and the protein was separated and purified by chitin-affinity chromatography and gel filtration chromatography. In addition, the activity of LDH is commonly used as a marker for the ability of dehydrins to rescue protein function during stress. We found that PicW1 can protect the enzyme activity of LDH which is better than BSA (a known protective protein) under freeze-thaw stress. However, in the high-temperature tests, the protective effect of PicW1 is similar to that of BSA and better than the negative control lysozyme (LZM).

## Materials and Methods

### Materials and Instruments

The chitin column (New England Biolabs, United States) and AKTA protein purification system were used for two-step protein purification. An ultrafiltration tube (Amicon ultra, United States) of 10 kDa millipore was used for protein concentration in the purification process. Liquid nitrogen was purchased from the Institute of Semiconductors, Chinese Academy of Sciences. The DU730 ultraviolet spectrophotometer (BECKMAN COULTER, United States) was used to measure the absorbance in the enzyme activity reaction. The protein concentration was measured using NanoDrop 2000c (Thermo Fisher Scientific, United States).

### Plasmid and Bacterial Strains

The *Picea wilsonii* dehydrin gene pTWIN1-PicW1 was given by Professor Jichen Xu (National Engineering Laboratory of Tree Breeding, Beijing Forestry University), and the recombinant plasmid, pRSF-Duet-LDH5, was given by Professor Fei Xiao (Peking University Fifth Clinical College of Geriatrics Key Laboratory). Both *E. coli* DH5α strain and *E. coli* BL21 (DE3) were purchased from TaKaRa Company.

### Protein Expression

**FIGURE 1:**
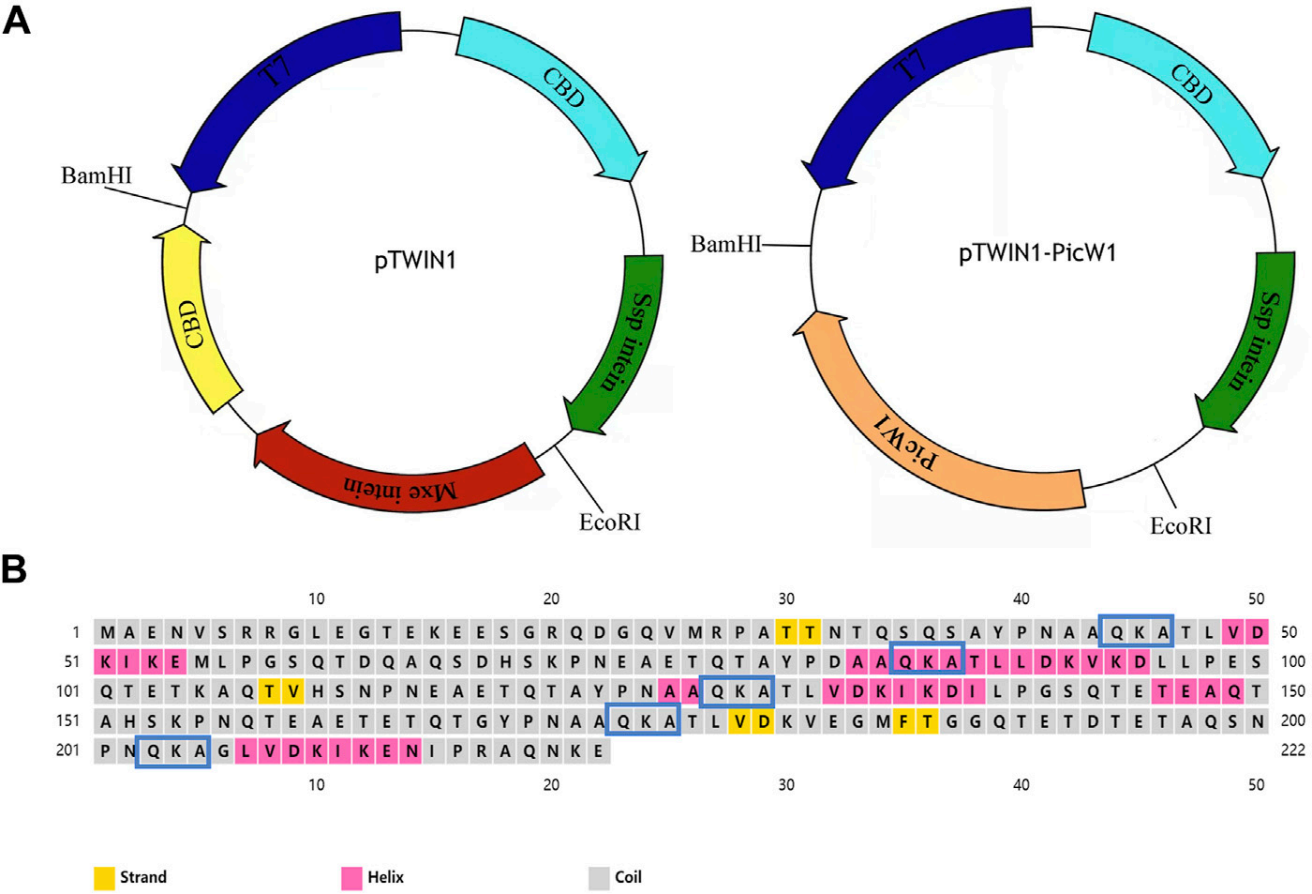
Prokaryotic expression vector pTWIN1 and secondary structures of PicW1. **(A)** CBD, chitin-binding domain; Mxe intein, *Mycobacterium* xenopi GyrA intein; Ssp intein, Synechocystis sp. DnaB intein gene; PicW1, the coding region of PicW1 gene; T7, T7promoter. **(B)** PicW1 has 222 amino acids, of which 5 K-segments start from the amino acid sequence QKA. Yellow squares represent the strand, pink squares represent the helix, and gray squares represent the coil.

*E. coli* BL21 (DE3) competent cells were transformed with the recombinant construct (Figure 1A). Ten milliliters of LB medium pH 7.0 containing ampicillin (100 μg/ml) were inoculated with a positive BL21 colony and then incubated for 12 h at 37°C with shaking at 220 rpm. One liter fresh LB medium containing ampicillin (100 μg/ml) was inoculated with the aforementioned cells and grown until the OD_600 nm_ reached 0.6–0.8 absorbance units (mid-log phase). Upon reaching the mid-log phase, protein expression was induced with isopropyl β-D-thiogalactoside (IPTG) at a concentration of 0.5 mM for 20 h at 16°C and 180 rpm. Then, the cells were harvested by centrifugation at 4,000 rpm for 30 min at 4°C. The pellet was saved and the supernatant was discarded.

### Protein Separation and Purification

The pellet was collected from 300 ml culture, lysed in 25 ml lysis buffer A containing 20 mM Tris-HCl pH 8.0, 500 mM NaCl, 1 mM EDTA, and 1 mM phenylmethylsulfonnmkyl fluoride (PMSF). The cells were sonicated on ice for 4 s 70–100 times spaced by about 6 s time interval to disrupt cell membranes. Then, the lysates were centrifuged for 30 min at 13,000 rpm at 4°C to separate soluble proteins from other cellular components. The clarified lysate was used for further purification.

Chitin-affinity chromatography was equilibrated with buffer A1 (20 mM Tris-HCl pH 8.0, 500 mM NaCl, and 1 mM EDTA). The clarified lysate (supernatant) was run onto a 3 ml chitin column which was followed by washing with 20-bed volumes of washing buffer B (20 mM Tris-HCl pH 8.0, 500 mM NaCl, 1 mM EDTA, 0.1% Triton-X100) to thoroughly remove non-specific proteins (O'Shea et al., 2015). The column was supplemented with three column volumes of cleavage buffer C (20 mM Tris-HCl pH 8.0, 500 mM NaCl, 1 mM EDTA, and 120 mM DTT) and placed on a rotary mixer. Then, the flow was stopped and the column was placed on a rotary mixer at 4°C for 48 h for the on-column cleavage of PicW1 protein. DTT was added every 6 h. The samples were collected in 6, 12, 18, 24, 30, 36, and 48 h and analyzed by 15% polyacrylamide gel electrophoresis on sodium dodecyl sulfate (SDS-PAGE). When the cutting was completed, the chitin matrix naturally settled through gravity sedimentation. The protein that flowed down was concentrated to within 2 ml in a 10 kDa concentration tube at 4,000 rpm, filtered through a 0.22 ultrafiltration membrane to remove impurities, and then prepared for the next purification operation.

The preliminarily purified protein solution was applied onto a Superdex 200 pg 10/300 GL column which was equilibrated with buffer D (20 mM Tris-HCl pH 8.0, 100 mM NaCl, 5 mM DTT) (O'Shea et al., 2015). Size exclusion was performed at 0.4 ml/min. The peaks of the target proteins were collected and concentrated to 10 mg/ml. The purified proteins were collected and tested through 18% SDS-PAGE. The LDH5 was prepared as previously described (Liu et al., 2017). The purified PicW1 and LDH5 were temporarily stored at 4°C. If long-term storage is required, 50% glycerol can be added to the protein and stored at −80°C.

### Lactate Dehydrogenase Protection Assay

The LDH protection assay was carried out in order to determine the protective role of the PicW1 protein. The activity of LDH was measured using the modified technique of Lin and Thomashow (1992). The negative control was LZM and the positive control was bovine serum albumin (BSA). All the proteins and reagents were dissolved in buffer E (10 mM phosphate buffer, pH 7.5). Different protein samples and LDH were mixed at the molar ratio of 0:1, 5:1, 10:1, and 20:1 into a 60 μl solution; the final concentration of LDH was 0.13 μM. The enzyme solution was divided equally into six tubes. For the cryoprotective assay, three tubes were subjected to freeze-thaw stress treatment and the other three tubes were left untreated. Freezing stress was imparted on the enzyme by five cycles of freezing in liquid nitrogen for 1 min and subsequent thawing in a water bath at 25°C for 5 min in the presence and absence of the PicW1 protein.

For the high-temperature stress assay, we set four temperatures which were 43, 49, 55, and 61°C, respectively, and prepared 60 μl enzyme solutions with the molar ratio (5:1) of different protein samples and LDH. The protein mixture solution was equally divided into six tubes; three copies were not processed, and the others were treated in the specific temperature water bath for 2 min, and then it was placed in the 25°C water bath for 5 min renaturation; the process was repeated three times.

Then, 5 μl enzyme solution was added to 2 ml of substrate (10 mM phosphate buffer pH 7.5, 0.1 mM NADH, and 7.5 mM pyruvate). The reduction of A340 in the reaction system was determined using a DU730 ultraviolet spectrophotometer within 3 min. The results were expressed as the LDH5 residual enzyme activity (%) relative to the activity prior to treatment. The untreated LDH activity was considered to be 100% at 25°C.

$$\text{L}\text{D}{\text{H}}_{5}\text{r}\text{e}\text{s}\text{i}\text{d}\text{u}\text{a}\text{l}\;enzyme\;activity\;\left(\%\right)\;=\;\;△A_{340b}/{△}_{340a}\;\times \;100\%$$

Here, ∆A_340a_ is the difference of A_340_ before the test sample is processed and ∆A_340b_ is the difference of A_340_ after the test sample is processed.

Three independent assays were performed, and SE was included. Values were the mean ± SD (n = 3). Student’s *t*-test analysis was performed between each sample of enzyme solution low-temperature protection experiment and 61°C high-temperature stress experiment.

## Results and Discussion

### Amino Acid Composition and Segment Characteristics of PicW1

In previous research, the full-length DHN gene PicW1 was isolated from *P. wilsonii* (Liu et al., 2014). Protein sequence analysis shows that PicW1 has a total length of 669 bp and encodes 222 amino acids. It is mainly composed of hydrophilic amino acids without cysteine(C) and tryptophan (W), with a high proportion of charged and polar amino acids and a low proportion of non-polar and hydrophobic residues (Table 1). According to Figure 1B, PicW1 is mainly composed of coil, which is normal for an IDP. DHNs contain at least one K-segment and as many as eleven (Jean-Marie et al., 2008), while PicW1 has five K-segments. Four K-segments are in the helical region predicted by the secondary structure, but only the second K-segment and the fourth K-segment form a relatively complete helical structure. The K-segment could form α-helices under abiotic stress to help stabilize membranes and proteins (Eriksson et al., 2011; Matthew et al., 2015; Mouillon et al., 2008). Some studies showed that the K-segment played a major role in the protection of enzymes at low temperatures, and prevented harmful changes in the secondary and tertiary structure of proteins (Yang et al., 2015). The truncated K-segment experiment showed that ERD10, RcDHN 5, and TaDHN -5 decreased the low-temperature protection of LDH (Peng et al., 2010; Cedeno et al., 2017). In addition, the lipid vesicle binding analysis of the three K-segments deletion derivatives of maize DHN1 proved that the α-helical conformation of the K-segment was essential for the binding of DHN to anionic phospholipid vesicles (Hara et al., 2011). The K-segment of wheat DHN-5 (YSK2) protected *E. coli* exposed to various stresses by preventing protein aggregation (Clarke et al., 2015).

**TABLE 1:** Protein and amino acid properties analysis.

Pro	Analysis			Amino acids (% by frequency)				
	L	MW	pI	Polar	Charged	Hydrophobic	Basic	Acidic
PicW1	222	24.1	4.9	35.43	29.60	23.77	11.21	15.25

L = length; MW = molecular weight; pI = isoelectric point; polar = NCQSTY; charged = RKHYCDE; hydrophobic = AILFWV; basic = KR; acidic = DE.

### Protein Expression and On-Column Purification by Affmity Chromatography

**FIGURE 2:**
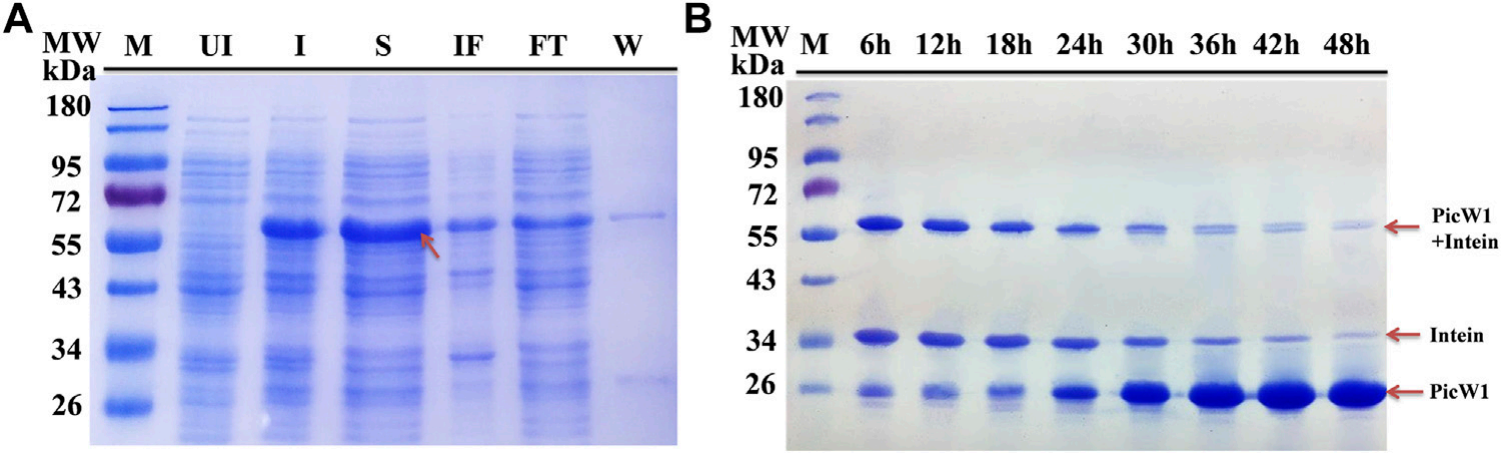
Purification of recombinant PicW1 by chitin-affinity chromatography. **(A)** SDS-PAGE analysis of the purification of PicW1-intein expressed in *E. coli* BL21 (DE3) cells. Lane 1, Marker = M; Lane 2, Un-induced BL21 cells lysate = UI; Lane 3, IPTG-induced BL21 cells lysate = I; Lane 4, Cell supernatant = S; Lane 5, Insoluble fraction = IF; Lane 6, Flow through = FT; Lane 7, wash with buffer B =W. Protein extraction along with intein tag at a range of about 55 kDa. **(B)** SDS-PAGE of PicW1 purification process using the chitin column. Lane1, marker; Lane 2 to Lane 9: 6, 12, 18, 24, 30, 36, 42, and 48 h represents the purification time.

The constructs used in the study are shown in Figure 1A. We selected *E. coli* as the heterologous expression system of Picea dehydrin gene, and the expressed protein can play its protective function (Yin et al., 2007). Some studies have shown that wheat dehydrin DHN-5, *Ammopiptanthus mongolicus* dehydrin AmCIP, and *Arabidopsis thaliana* dehydrins RAB18, LTI29, LTI30, and COR47 were heterologously expressed in the prokaryotic expression system, and the protein size and activity are correct (Svensson et al., 2000; Brini et al., 2010; Shi et al., 2016). The presence of the expressed protein in the supernatant of lysates from the transformant cells was examined by SDS-PAGE gel electrophoresis. Figure 2A indicated that induced cells produced a band with a predicted molecular weight of CBD-intein-PicW1 at around 55 kDa. The recombinant protein mainly existed in the supernatant after cell disruption and a small amount in insoluble components. We purified the PicW1 protein by affinity chromatography *via* intein-mediated purification using an affinity chitin-binding tag system; a novel protein purification system which utilizes the inducible self-cleavage activity of protein splicing elements (termed inteins) to separate the target protein from the affinity tag. PicW1-intein-CBD can specifically bind to chitin beads. Intein could self-cleavage in the presence of the reducing agent DTT, so that the target protein PicW1 and intein-CBD will be separated. Intein-CBD was adsorbed on the chitin beads, and the target protein PicW1 existed in the cutting buffer and was finally eluted by the gravity flow. As shown in Figure 2B, most of the target proteins were completely cleaved at 48 h.

**FIGURE 3:**
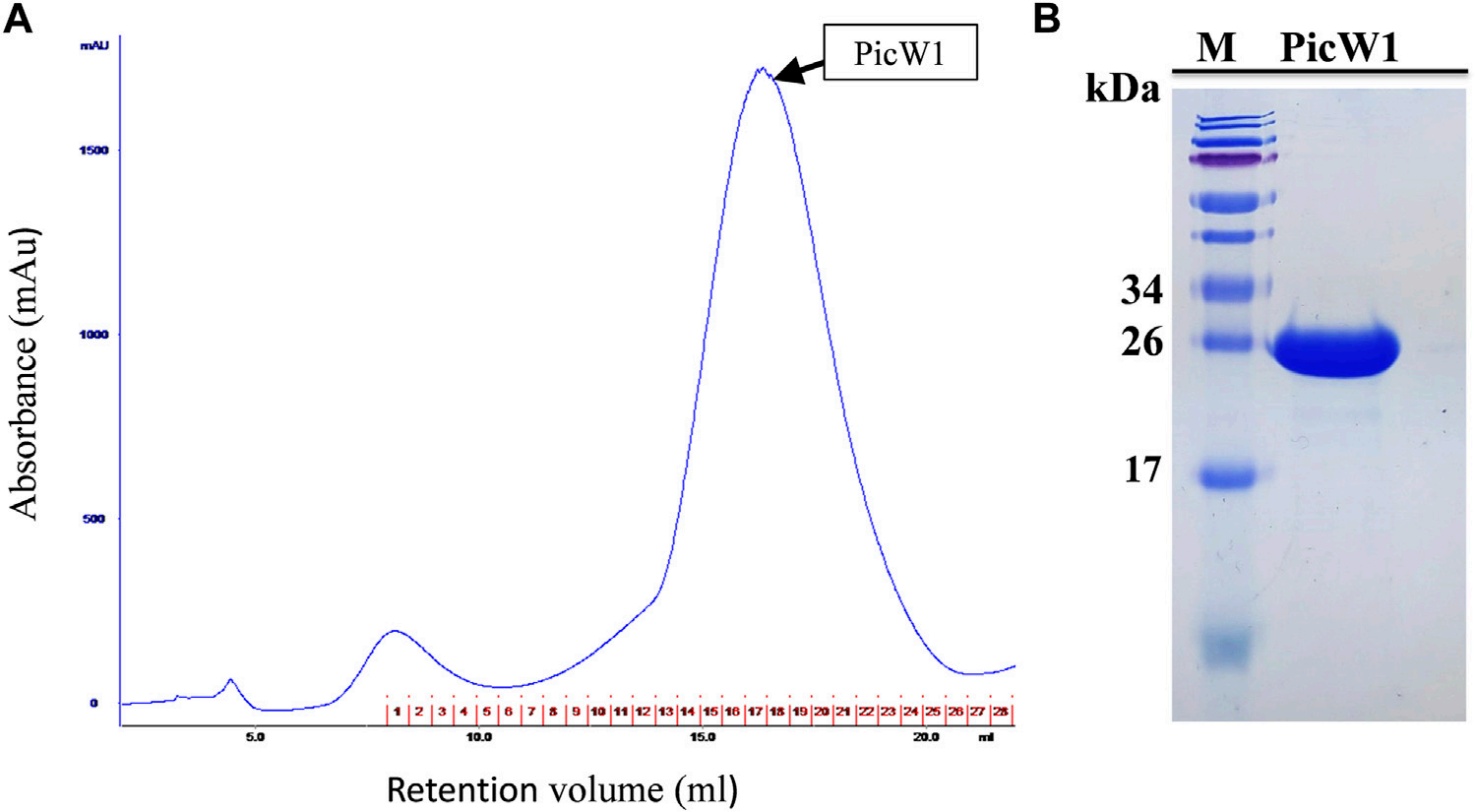
Purification of PicW1 by size-exclusion chromatography. **(A)** Gel filtration chromatography of PicW1 on a Superdex 200 pg 10/300 GL column. Elution was monitored at 280 nm at a flow rate of 0.4 ml/min. The red numbers represent the tubes of protein components collected using the AKTA collector. **(B)** SDS-PAGE analysis of “PicW1” purified using the Superdex 200 pg 10/300 GL column. Lane 1, Marker = M; Lane 2, protein components at peak value of gel filtration curve.

PicW1 was further purified using the AKTA protein purification system of the Surperdex 200 pg 10/300 GL column, with a major and symmetrical peak at 17 ml. The gel filtration curve of the Superdex 200 preparative column showed that PicW1 was a monomer that matched with the molecular weight value calculated from its primary sequence (Figure 3A). The PicW1 components were collected from the AKTA system for crystallization and subsequent functional tests. As shown in Figure 3B, the target protein at the peak of the curve was identified using 18% SDS-PAGE, with a molecular weight of about 24 kDa. The final yield was ∼20 mg PicW1 per liter of culture medium.

### PicW1 Protect the Enzyme Lactate Dehydrogenase Under Freezing Stress and High Temperature

**FIGURE 4:**
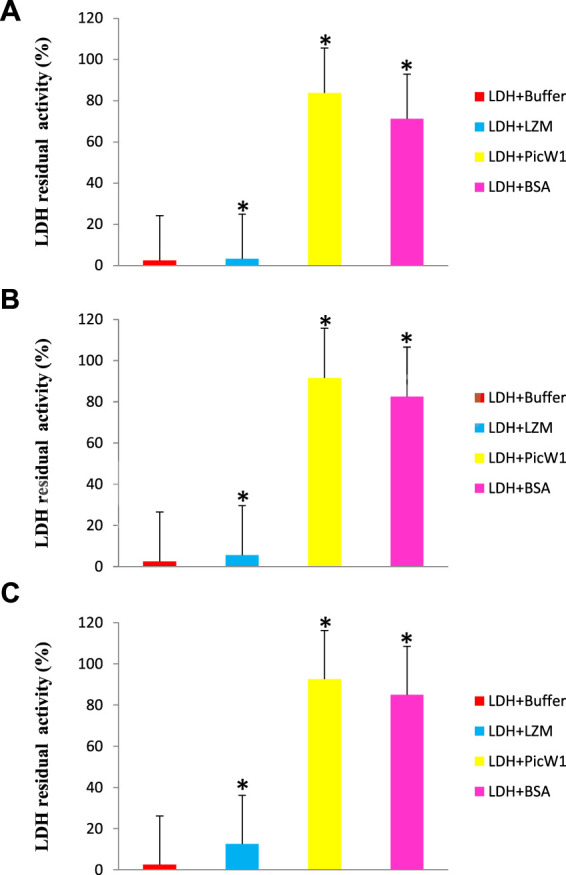
PicW1 protected LDH activity from freeze-thaw stress. Different molar ratios of LDH solutions in the presence of BSA, LZM, and purified recombinant proteins PicW1 were subjected to freeze-thaw stress for the specified times **(A)**, 5:1; **(B)**, 10:1; **(C)**, 20:1 represent the molar ratio of added protein to LDH; the buffer group only has LDH. LDH activity was measured at each particular time point. The results are expressed as the LDH activity (%) relative to the activity before treatment. Three independent assays were performed, and SE was included. Values are the mean ± SD (n = 3). Significant differences in the LDH enzyme activity retention are indicated as *p < 0.05, which were evaluated with a *t*-test (*t*-test was performed on the protective effect of every two proteins of LZM, PicW1, and BSA on LDH).

Because LDH loses its activity during freezing and high-temperature stress, we select this enzyme to evaluate the protective effect of PicW1. The activity of LDH before the experiment was considered as 100%. The enzyme LDH lost its activity completely upon repeated 1 min freezing and 5 min thawing at -196°C for five times (Figure 4A). In contrast, LDH activity retained to nearly 84% in the presence of 0.65 µM (5:1) PicW1, which was higher than that with BSA (71%; Figure 4A). We also found approximately 92% LDH activity was retained when 1.30 µM (10:1) and 2.6 µM (20:1) PicW1 were added, which was higher than that with BSA (84%; Figure 4B, Figure 4C). The increased concentration of purified PicW1 increased the percentage of LDH activity retained after freeze-thaw assay (Figure 4).

Many cold sensitive enzymes are oligomers, including LDH, β-glucosidase (bglG), and catalase. Low-temperature stress makes subunit denaturation unable to combine correctly, and then irreversibly reduces the enzyme activity (Goyal et al., 2005). Brini et al. showed that wheat DHN-5 increased the activity and thermal stability of fungal bglG and glucose oxidase (GOD/POD) (Brini et al., 2010). However, PicW1 contains five K-segments and is different from the typical K-segment sequence. It has been reported that the deletion of K-segment of wheat dehydrin WZY2 leads to the reduction of low-temperature protection, indicating that these segments are involved in low-temperature protection (Yang et al., 2015). It is speculated that the hydrophobic side of picw1 may interact with the lipid components of the biofilm or combine with the hydrophobic region of the denatured part of the protein to form a protective layer to prevent the denatured macromolecular substances from further denaturation and polymerization (Rahman et al., 2010). Cor15 may form a random curl in the low-temperature protection experiment, which means that except for the K-segment, the irregular curl improves the structural flexibility and may play an important role in the low-temperature protection of cold-sensitive enzymes (Ingram and Bartels, 1996). Picw1 protein contains a large number of irregular curls, which may play a positive role in freeze-thaw stress. In conclusion, PicW1 has cryoprotective activity on LDH and has a more significant protective effect than BSA (a known protective protein) from the deleterious effects of freeze-thaw treatment. These findings serve to not only further define the molecular characteristics and possible functions of PicW1, but also add to the pool of evidence that supports a role for DHN proteins in cold tolerance.

**FIGURE 5:**
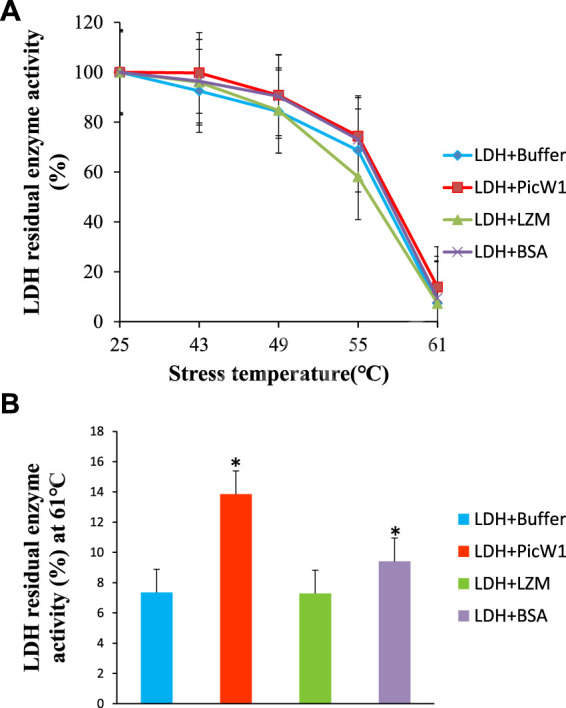
Protective effect of PicW1 protein on LDH activity under high-temperature stress. **(A)** LDH solution was cultured in high-temperature water bath (43, 49, 55, and 61°C) for a specific time in the presence of PicW1, BSA, and LZM. The results were expressed as LDH residual enzyme activity (%) relative to the activity before stress. Error bar represents ±SEM from at least three replicates. **(B)** Heat stress treatment of LDH in the presence and absence of PicW1 at 61°C. Significant differences in the LDH enzyme activity retention are indicated as *p < 0.05, which were evaluated with a *t*-test (*t*-test was performed on the protective effect of every two proteins of LZM, PicW1, and BSA on LDH).

The effect of PicW1 in protecting LDH activity during high-temperature stress was also evaluated. Similarly, BSA was selected as a positive control, and LZM was used as a negative control. As shown in Figure 5A, the enzyme activity of LDH decreased with the increase of stress treatment temperature. The LDH enzyme activity in the presence of PicW1 was higher than that in the LZM experimental group and blank control group at each temperature, but it was only slightly better than that in the BSA group at 43–55°C. The LDH enzyme almost completely lost its activity after three heat stress cycles at 61°C, only 7%. A minimum concentration of 0.65 µM PicW1 was found to be sufficient to retain 14% of the enzymatic activity under high-temperature stress (61°C) than the 9% of 0.65 µM BSA. The student t-test of 61°C data (Figure 5B) showed that the protective effect of PicW1 on LDH was better than that of other experimental groups, including the BSA group. The cryoprotection of dehydrin on LDH has been widely studied. The dehydrated protein is more effective than small molecules (such as sucrose) or other proteins (such as BSA) in protecting LDH activity from freeze-thaw damage (Hughes and Graether, 2011). The protective effect of BSA is generally considered as non-specific ones of high protein concentrations (Abriata et al., 2016; Huang et al., 2022). Although the protective effect of dehydrins on other proteins is mainly observed at low temperature, there are relatively few studies on high-temperature stress. Previously, LEA protein COR15 could protect the activity of LDH, but the temperature study was limited to 43°C (Nakayama et al., 2008). In addition, DHN-5 could significantly improve the activity and thermal stability of bglG at 50 and 70°C (Brini et al., 2010). Here, we propose a heat stress assay at 43–61°C, which shows that PicW1 can effectively protect LDH under high-temperature treatment up to 61°C.

**FIGURE 6:**
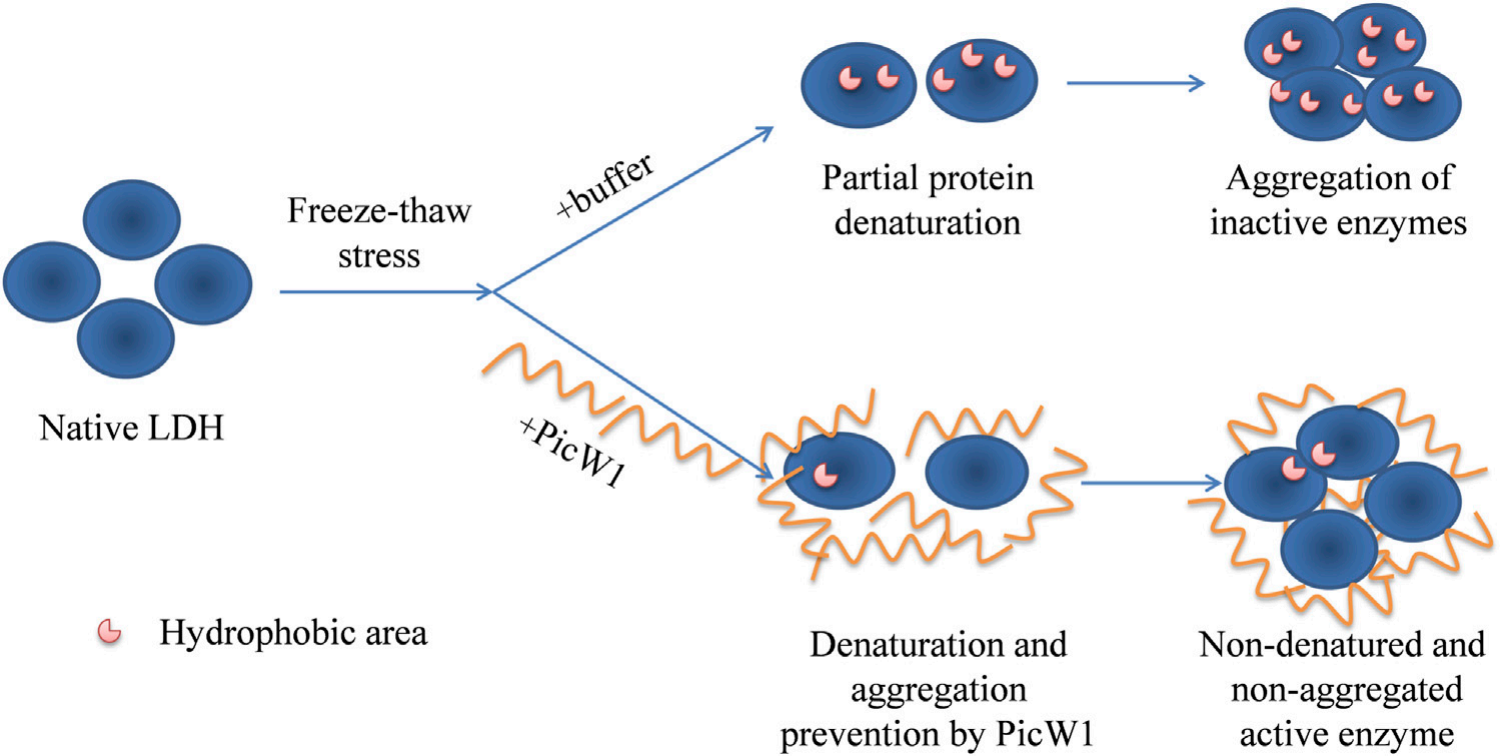
Putative mechanisms for cryoprotective activity of PicW1. When LDH is frozen and thawed, hydrophobic patches appear on the surface of the proteins, and then hydrophobic interaction is generated between the patches. PicW1 acts as a shielding molecule to interfere with the hydrophobic interaction between LDHs. Finally, LDH maintains a more natural state.

Dehydrin interacts with the lipid components of biofilm or combines with the hydrophobic region of protein denatured part to form a protective layer, prevent the further denaturation and polymerization of denatured macromolecules, and help plants stabilize cell membrane and proteins under stress. Immunolocalization studies showed that dehydrin accumulated more in the plasma membrane or areas rich in the membrane structure, around lipids and proteins. For example, the dehydrin lti29 gene was overexpressed in Arabidopsis. During cold acclimation, the LTI29 of transgenic plants was transferred from cytoplasm to near membrane, and the survival rate of plants under freezing stress was improved (Puhakainen et al., 2004). DHN can act as a partner for other proteins to help them fold correctly and prevent them from aggregating under heat or cold stress (Hara et al., 2001). However, the chaperone activity of traditional proteins can also form specific complexes with target proteins through the interaction of hydrophobic plaques (Ellis, 2004). Therefore, DHN is more like a barrier between molecules, preventing the aggregation of denatured proteins. We have compared the activity of the PicW1 protein with another known protectant like BSA. The results showed that PicW1 protein could protect the LDH activity better than BSA under freeze-thaw stress. In addition, the higher the concentration of PicW1, the greater the protective effect on LDH. It may be imagined that PicW1 is like a “wall” that prevents the denaturation and aggregation of LDH. The more the “walls”, the more LDH activity is retained (Figure 6).

## Summary

Among the group of LEA proteins, dehydrins play a major role in protection during abiotic stress. DHNs are known as stress-responsive proteins that are up-regulated in plants during stress such as drought, cold, and salinity (Hara et al., 2005). In this work, we have highly expressed cold acclimation protein PicW1 from *Picea wilsonii* in *E. coli*. We also proved that PicW1 has superior low-temperature protection on LDH. These approaches should be applicable to other plant dehydrins. The success in PicW1 expression and purification will facilitate future structural studies to understand how it works in stress resistance. In the future, we will explore the mechanism of PicW1 in depth.

## Data Availability

The original contributions presented in the study are included in the article/Supplementary Material; further inquiries can be directed to the corresponding authors.
